# TET enzyme driven epigenetic reprogramming in early embryos and its implication on long-term health

**DOI:** 10.3389/fcell.2024.1358649

**Published:** 2024-08-01

**Authors:** Ty Montgomery, Kyungjun Uh, Kiho Lee

**Affiliations:** ^1^ Division of Animal Sciences, University of Missouri, Columbia, MO, United States; ^2^ Futuristic Animal Resource and Research Center, Korea Research Institute of Bioscience and Biotechnology, Cheongju-si, Republic of Korea

**Keywords:** TET family enzymes, epigenetics (DNA methylation), DNA methylation (5mC), fertilization, DNA demethylation during development

## Abstract

Mammalian embryo development is initiated by the union of paternal and maternal gametes. Upon fertilization, their epigenome landscape is transformed through a series of finely orchestrated mechanisms that are crucial for survival and successful embryogenesis. Specifically, maternal or oocyte-specific reprogramming factors modulate germ cell specific epigenetic marks into their embryonic states. Rapid and dynamic changes in epigenetic marks such as DNA methylation and histone modifications are observed during early embryo development. These changes govern the structure of embryonic genome prior to zygotic genome activation. Differential changes in epigenetic marks are observed between paternal and maternal genomes because the structure of the parental genomes allows interaction with specific oocyte reprogramming factors. For instance, the paternal genome is targeted by the TET family of enzymes which oxidize the 5-methylcytosine (5mC) epigenetic mark into 5-hydroxymethylcytosine (5hmC) to lower the level of DNA methylation. The maternal genome is mainly protected from TET3-mediated oxidation by the maternal factor, STELLA. The TET3-mediated DNA demethylation occurs at the global level and is clearly observed in many mammalian species. Other epigenetic modulating enzymes, such as DNA methyltransferases, provide fine tuning of the DNA methylation level by initiating *de novo* methylation. The mechanisms which initiate the epigenetic reprogramming of gametes are critical for proper activation of embryonic genome and subsequent establishment of pluripotency and normal development. Clinical cases or diseases linked to mutations in reprogramming modulators exist, emphasizing the need to understand mechanistic actions of these modulators. In addition, embryos generated via *in vitro* embryo production system often present epigenetic abnormalities. Understanding mechanistic actions of the epigenetic modulators will potentially improve the well-being of individuals suffering from these epigenetic disorders and correct epigenetic abnormalities in embryos produced *in vitro*. This review will summarize the current understanding of epigenetic reprogramming by TET enzymes during early embryogenesis and highlight its clinical relevance and potential implication for assisted reproductive technologies.

## 1 Introduction

Mammalian embryo development is initiated by the fertilization event. The haploid genome of oocyte and sperm are combined after fertilization and the genomes undergo origin-specific remodeling of their epigenome. After the remodeling, embryonic cells, i.e., blastomeres, start to possess unique epigenomic make-ups at the time of lineage specification. Proper remodeling of the epigenome is necessary for normal development and the dynamic changes are governed by a series of finely orchestrated events. The epigenetic reprogramming that embryos undergo includes changes to chromatin structure due to the remodeling of histone modifications and DNA methylation. This first wave of reprogramming initiates the change from germ cell specific epigenetic marks to embryo specific marks. Also, lineage-specific epigenetic markers are established as embryonic cells differentiate into fetal and placental lineages.

Global reprogramming of DNA methylation marks in early embryos has been intensively studied because disruption to the remodeling can have severe impact on development. Upon fertilization, a rapid decrease in DNA methylome is observed in multiple species, including humans (Dean et al., 2001; Santos et al., 2002; Guo H. et al., 2014). This demethylation event organizes the epigenetic structure of the embryonic genome prior to zygotic genome activation. Recent studies point out that the DNA demethylation is orchestrated by the ten-eleven translocation (TET1/2/3) enzyme family by oxidizing 5-methylcytosine (5mC) into 5-hydroxymethylcytosine (5hmC) (Tahiliani et al., 2009). Then, the 5hmC are ultimately converted into non-methylated cytosine (He et al., 2011; Shen et al., 2013). Demethylation of the genome is important for embryo survival and normal development, but the specific mechanisms underlying the remodeling are still under investigation (Wossidlo et al., 2011).

Disruption of the *TET* family during early development increases frequency of infertility, neurological defects, and cancer development in animal models (Dawlaty et al., 2011; Dawlaty et al., 2013; Zhang et al., 2013; Fahrner, 2023). The ablation of Tet1 causes neurological defects in spatial learning and short-term memory in mouse models (Rudenko et al., 2013; Zhang et al., 2013). Mouse models lacking functional Tet2 have an increased propensity of hematopoietic cell lineage malignancies (Li et al., 2011). Dysregulation of TET1 or TET2 is also associated with diseases in the clinic (Good et al., 2017; Cao et al., 2019; Jiang, 2020). The mutation or silencing of TET3 causes the onset of Beck-Farhner syndrome, an autosomal dominant epigenetic disorder, in humans (Fahrner, 2023). Clinical data and animal studies highlight conserved roles of the TET family in mammals and their importance for normal development.

In this review, we summarize and discuss recent findings underlying mechanisms regulating epigenetic reprogramming upon fertilization, particularly related with DNA methylation dynamics during preimplantation development in mammals. The mechanism of the reprogramming events will be outlined while emphasizing species specific characteristics. Epigenetic disorders found in patients that are rooted to dysregulated genes involved in the reprogramming process will also be discussed.

## 2 Epigenetic marks derive successful development

Biochemists and cytologists from the late 19th century were the first to observe that a DNA and protein complex existed in the cell’s nucleus, and the complex was named ‘chromatin’ by Walther Flemming in the 1880’s (Olins and Olins, 2003; Deichmann, 2015). Although the role of chromatin was not clear, it was suggested to influence gene expression without changing gene structure. Research on the modification of chromatin structure demonstrated that their conformation is linked to the expression of a gene without changing the DNA sequence, paving the concept of epigenetics.

The family of proteins called histones are the primary protein component of chromatin (Li, 2002). An octamer of these small and highly conserved proteins is associated with approximately 200 base pairs of DNA to form a nucleosome (Li, 2002). The lysine, arginine, and serine residues on the amino-terminal tail of histones can be modified to influence the accessibility of chromatin, thus highlighting the impact of histone tails on gene expression (Li, 2002). For example, transcriptional activation is associated with methylation of lysine on histone H3 at the fourth residue (H3K4me3), H3K36me, and H3K79me (Miao and Natarajan, 2005; Morgan et al., 2005). Alternatively, transcription suppression is accompanied by H3K9me3, H3K27me3, and H4K20me3 marks (Miao and Natarajan, 2005; Morgan et al., 2005). Other post-translational modifications such as phosphorylation, acetylation, and ubiquitylation of histones can also regulate gene activity (Li, 2002). Chromatin structure is also influenced by direct methylation of the nucleic acids within the DNA strand. The 5-methylcytosine (5mC) was first observed by Johnson and Coghill in 1925 (Johnson and Coghill, 1925); however, it wasn’t until 1975 when DNA methylation was labeled as an epigenetic mark (Holliday and Pugh, 1975; Riggs, 1975). After identifying presence of 5mC in mammalian, insect, and plant DNA in 1950, their distribution patterns on the genome were confirmed 4 years later (Wyatt, 1950; Sinsheimer et al., 1954). The 5mC marks were specifically detected before guanine (CpG dinucleotides), rather than being randomly distributed throughout the genome (Sinsheimer et al., 1954). In mammalian cells, DNA methylation is predominantly found at CpG dinucleotides, and methylation of CpG dinucleotides in the promoters of genes is typically associated with epigenetic silencing of gene transcription (Weber et al., 2007; Meissner et al., 2008; Jones, 2012). Histone modification and DNA methylation mechanisms are linked together (Li, 2002; Morgan et al., 2005) as DNA methylation marks are added to the genome by DNA methyltransferases (DNMT) and their activity are highly correlated to the local chromatin states that are controlled by histone modifications (Burgers et al., 2002).

Epigenetic marks are in general maintained consistently during cell division, i.e., mitosis. Interestingly, during preimplantation development, highly methylated genomes, inherited from germ cells, are dramatically demethylated until blastocyst stage except for imprinting control regions (ICRs) and certain retrotransposons (Borgel et al., 2010; Smallwood et al., 2011; Kobayashi et al., 2012; Smith et al., 2012; Smith et al., 2014). The maternal and paternal genomes exhibit different rates of DNA demethylation during preimplantation development and distinct demethylation pathways are involved; the maternal genome is passively demethylated, whereas the paternal genome is actively demethylated (Borgel et al., 2010; Smallwood et al., 2011; Kobayashi et al., 2012; Smith et al., 2012; Smith et al., 2014). Specifically, the paternal pronuclei are demethylated by the TET3 enzymes while the maternal pronuclei are protected by the protein STELLA (Wossidlo et al., 2011; Nakamura et al., 2012). Later in development, DNA methylation marks of the genes that are critical for pluripotency of embryos are re-established by *de novo* methylation (Li et al., 2007; Meissner, 2010). Although the key sequential reprogramming events are conserved across most mammals, species specific differences such as degree of demethylation and timing of onset in *de novo* methylation have been reported (Young and Beaujean, 2004; Hou et al., 2007). Mechanistic actions driving the reprogramming of gamete genome after fertilization have not been fully elucidated.

## 3 DNA methylation as the main epigenetic marker and its regulation

In mammalian cells, the presence of methylated CpG dinucleotides, specifically 5-methylcytosine (5mC), in the promoter regions is in general interpreted as silencing of genes (Weber et al., 2007; Meissner et al., 2008; Jones, 2012). Non-CpG methylation has recently been identified in other cell types such as oocytes, embryonic stem cells (ESCs), and neurons (Lister et al., 2009; Xie et al., 2012; Lister et al., 2013; Shirane et al., 2013). Methylation of cytosine preceding cytosine (CpC), thymine (CpT), and adenine (CpA) accounts for approximately 15% of cytosine DNA methylation (Ziller et al., 2011) but the epigenetic role of such methylation marks remains to be identified. The role of non-CpG methylation during early embryo development is largely unknown. Interestingly, an average of non-CpG methylation level, mostly CpA bases, is greater in oocytes (∼3%) than any other stage of development, including post-implantation (∼1%) (Li C. et al., 2018). There is little evidence it has a widespread impact on gene expression during embryogenesis. DNA methylation marks are established by two major *de novo* methyltransferases, DNA methyltransferase 3A (DNMT3A) and DNA methyltransferase 3B (DNMT3B) (Okano et al., 1998; Okano et al., 1999). A catalytically in-active DNMT, DNMT3L, is also involved in *de novo* methylation specifically in germline by stimulating activities of DNMT3A and DNMT3B through direct interaction (Suetake et al., 2004; Ooi et al., 2007). Maintenance of DNA methylation marks is led by DNA methyltransferase 1 (DNMT1). Since DNA replication occurs in semiconservative manner, newly synthesized complimentary sequence on daughter DNA strands lacks DNA methylation. Because DNMT1 preferably bind to hemi-methylated CpG dinucleotides, it functions as a key regulator, which maintain symmetrical DNA methylation levels throughout cell divisions (Bestor et al., 1988; Bestor, 1992). A depletion of DNMT1 results in the passive demethylation of the genome as DNA methylation is diluted after DNA replication (Holliday and Pugh, 1975; Hermann et al., 2004; Nishiyama et al., 2013).

The role and maintenance of 5mC have been intensively studied. Other modifications to the cytosine have been reported; however, their functions are largely unknown. For instance, the hydroxylated form of 5mC, 5-hydroxymethylcytosine (5hmC) was first reported in 1972; and it was originally reported that 5hmC accounted for ∼15% of total cytosines in DNA isolated from brain tissues of rat, mouse, and frog (Penn et al., 1972). The presence of 5hmC in mammalian DNA could not be confirmed by other studies until it was robustly detected in the mouse cerebellum and ESCs by two research groups in 2009 (Kriaucionis and Heintz, 2009; Tahiliani et al., 2009). The 5hmC residue accounted for 0.6% of the total nucleotides in mouse Purkinje neurons and 0.03% of the total nucleotides in mouse ESCs (Kriaucionis and Heintz, 2009; Tahiliani et al., 2009). Another study identified 5hmC in various mouse and human tissues with high levels in the central nervous system (Song et al., 2011). Other cytosine bases such as 5-formylcytosine (5fC) and 5-carboxycytosine (5caC) have also been reported. However, the role of the bases is not clearly understood.

Discovery of the diverse cytosine bases prompt investigations on how the bases are converted. Identifying enzymes responsible for converting 5mC into 5hmC was inspired by the production of base J (β-D-glucosyl hydroxymethyluracil) in trypanosomes (Borst and Sabatini, 2008). Base J is a modified thymine associated with gene silencing, similar in function to 5mC in mammals and is synthesized by the hydroxylation of a methyl group of thymine (Borst and Sabatini, 2008). It was suggested enzymes JBP1 and JBP2 catalyzed the hydroxylation of the methyl group of thymine as a part of the 2-oxoglutarate (2OG)- and Fe(II)-dependent oxygenase superfamily (Yu et al., 2007; Cliffe et al., 2009). Following the studies, Tahiliani et al. (2009) identified the ten-eleven translocation (TET) proteins as mammalian homologs of the trypanosome proteins JBP1 and JBP2 and demonstrated that the TET enzymes are 2OG- and Fe(II)-dependent enzymes that catalyze the conversion of 5mC to 5hmC (Tahiliani et al., 2009). Subsequent studies revealed that 5hmC can be further oxidized to 5-formylcytosine (5fC) and 5-carboxycytosine (5caC) by the TET enzymes (He et al., 2011; Ito et al., 2011). The discovery and conversion of the three 5mC derivatives suggested a new demethylation mechanism orchestrated by the TET enzymes. Recent studies found that thymine DNA glycosylase (TDG), an enzyme mediating base excision repair of DNA, has direct activity with 5fC and 5caC, implying that these two cytosine bases are intermediates of the active demethylation process (He et al., 2011; Shen et al., 2013). In addition, conversion of 5mC to 5hmC by the TET enzymes may aid in the acceleration of passive demethylation because affinity of DNMT1 towards 5hmC is much lower than for 5mC in hemi-methylated DNA strands, thus preventing the addition of 5mC to a newly synthesized strand (Hashimoto et al., 2012; Ji et al., 2014). The discovery of the DNA demethylation pathways greatly expanded our understanding of how DNA methylation marks are regulated. Conventionally, it was hypothesized that the lack of DNMT would derive DNA demethylation in cells; however, the theory could not explain some of rapid changes in DNA methylation marks seen in cells especially during embryogenesis. The TET enzyme-mediated DNA demethylation pathway offers more detailed explanation to the dynamic changes in epigenetic marks and provide clues for successful cellular reprogramming and mechanistic understanding to certain diseases associated with aberrant DNA methylation.

## 4 DNA methylation during pre-implantation development

The establishment of DNA methylation marks has been extensively studied and its impact on gene regulation is generally understood. Yet, mechanistic pathways of active DNA demethylation and its influence on gene regulation are still under investigation. Active DNA demethylation in mammalian tissues is mediated by the TET enzymes which oxidize the 5mC mark into 5hmC (Kriaucionis and Heintz, 2009; Tahiliani et al., 2009). This discovery quickly advanced our understanding of epigenetic regulation during early embryo development in mammals. Following fertilization, genome wide demethylation process rapidly erases DNA methylation marks, which were inherited from germ cells, and starts to establish embryonic DNA methylation patterns.

Amino acid sequences of TET family are highly conserved among species and share key domains in all species (Table 1). The conserved sequences highlight the importance of TET enzymes in leading epigenetic reprogramming for successful embryo development in mammals. Three TET enzymes, TET1/2/3, are differentially expressed throughout the stages of preimplantation development and responsible for the formation of 5hmC in different stages (Tahiliani et al., 2009; Ito et al., 2010). The TET3 protein is detected in oocytes and zygotes and highly expressed in neurons (Iqbal et al., 2011; Wossidlo et al., 2011; Perera et al., 2015; Yu et al., 2015). The Tet1 and Tet2 proteins are highly expressed in the inner cell mass (ICM) of blastocysts, ESCs, and PGCs (Ito et al., 2010; Vincent et al., 2013). The *TET2* is also highly expressed in hematopoietic stem cells (HSCs) and essential in hematopoiesis, including HSCs self-renewal and lineage commitment (Solary et al., 2014). A *Tet1* knockout (KO) resulted in reduced birth weight and subfertility in both male and female mice, but the modification did not lead to embryo lethality (Dawlaty et al., 2011; Yamaguchi et al., 2012). The mild impact of *Tet1* KO on development has been thought to be due to functional redundancy between Tet1 and Tet2, as they possess overlapping expression patterns in ESCs and HSCs (Costa et al., 2013; Zhao et al., 2015). A double KO of *Tet1* and *Tet2* did not cause any visible abnormalities in mice while its influence on epigenome remains unclear (Wang et al., 2013) Homozygous *Tet3* KO resulted in neonatal lethality (Wang et al., 2013; Tsukada et al., 2015), and the loss of maternal Tet3 caused embryonic sub-lethality (Gu et al., 2011).

**TABLE 1:** Comparison of the human TET family proteins to domestic species orthologs. Length (number of amino acids) of human TET family member denoted on the left. The percentage of identity shared between each species ortholog and the human protein.

	Length of human protein (AA)	Mouse	Pig	Cattle	Sheep
		Identity (%)	Identity (%)	Identity (%)	Identity (%)
TET1	2165	55.0	77.5	77.5	77.9
TET2	2002	60.1	84.7	82.4	82.8
TET3	1795	89.3	91.6	91.4	91.3

**FIGURE 1:**
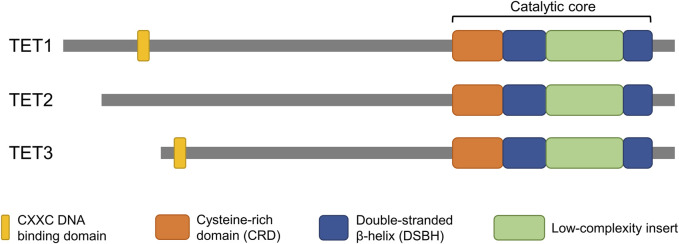
Domain structure of TET proteins. The catalytic domain, which is conserved in all members of the TET family, is located at the C-terminus. The CXXC domain, which confers DNA binding properties, is located at the N-terminus of TET1 and TET3 but is not present in TET2. The structural similarities highlight potential overlapping function of the genes.

The conversion of 5mC to 5hmC is directly mediated by the catalytic domain of TET enzymes. The catalytic domain is located at the carboxyl terminal region of TET enzymes and consists of a cysteine-rich domain (CRD) and two double-stranded beta-helixes (DSBH) (Iyer et al., 2009; Tahiliani et al., 2009) (Figure 1). Two zinc fingers bind the CRD and the DSBH domain to each other to form a compact catalytic domain (Hu et al., 2013). While the catalytic domain is present in all members of the TET family (TET1/2/3), the CXXC domain, which is located at the amino terminal region, is not present in TET2. The CXXC domain mediates specific binding of TET enzymes to DNA containing CpG dinucleotides (Xu et al., 2011; Xu et al., 2012). TET1 and TET3 have a tendency to bind preferentially to CpG-rich promoter regions, and the DNA binding property of the CXXC domain is thought to confer this property (Williams et al., 2011; Wu et al., 2011; Xu et al., 2012). The CXXC domain of an ancestral TET2 was lost through chromosomal inversion during evolution and then became a separate gene, IDAX (also called CXXC4) (Ko et al., 2013). In contrast to TET1 and TET3, 5hmC regulated by TET2 is abundant in gene body and exon regions rather than in promoter regions (Huang et al., 2014). The structural similarities, especially between TET1 and TET3, indicate their overlapping function (Figure 1). However, it is unclear why the TET family genes are differentially expressed during early embryo development.

### 4.1 Active demethylation of paternal genome

Upon fertilization, the paternal genome exhibits higher methylation level than maternal genome (Bestor, 2000). In mice, the level of CpG methylation of the mature sperm is between 80% and 90%, while mature oocytes show relatively lower-level levels (∼40%) (Popp et al., 2010). The highly methylated paternal genome is almost completely demethylated shortly after fertilization while demethylation of maternal genome occurs gradually as embryos divide (Oswald et al., 2000). The removal of methylation marks of paternal genome is almost completed before the onset of DNA replication at pronuclear stage 3 (PN3), suggesting existence of an enzyme-mediated active DNA demethylation although no “demethylase” was identified (Mayer et al., 2000; Oswald et al., 2000). Bisulfite sequencing was difficult to interpret especially in the paternal pronuclei, even though immunofluorescence assays clearly demonstrated a loss of 5mC at the same stage (Hajkova et al., 2008; Wossidlo et al., 2010). The discrepancy between bisulfite sequencing and immunofluorescence assay detection of methylation status was due to the formation of 5hmC. Specifically, both 5mC and 5hmC are read as cytosine in the conventional bisulfite sequencing, thus 5hmC cannot be discriminated from 5mC (Huang et al., 2010).

The discovery of TET enzymes and 5hmC in mature oocytes and zygotes provided clues to understand the active demethylation of paternal genome in zygotes (Gu et al., 2011). The TET3 enzyme mediates active demethylation by catalyzing the conversion of 5mC to 5hmC in paternal pronuclei (Gu et al., 2011; Iqbal et al., 2011; Wossidlo et al., 2011). The 5hmC signal is enriched in paternal pronuclei of zygotes (Tahiliani et al., 2009; Ito et al., 2010). Subsequently, the 5hmC is further oxidized to 5fC and 5caC by TET3 in zygotes (Inoue et al., 2011). Immunofluorescent staining of 5hmC has been the main technique to capture the level of 5hmC. Development of diverse molecular tools now allow for more detailed identification of 5hmC signals. Specifically, advanced sequencing methods such as TET-assisted bisulfite sequencing (TAB-Seq), oxidative bisulfite sequencing (oxBS), and TET-assisted pyridine borane sequencing (TAPS) have been developed for detection of 5hmC at base-resolution (Booth et al., 2012; Yu et al., 2012; Liu Y. et al., 2019).

The Tet3 enzyme is enriched in the paternal pronucleus where it catalyzes the oxidation of 5mC to 5hmC (Gu et al., 2011; Wossidlo et al., 2011). In the absence of Tet3, the level of 5mC remained constant in the paternal genome in zygotes (Gu et al., 2011). The Tet3 deficiency also impeded the demethylation of paternal alleles essential for embryo development and pluripotency, such as Oct4 and Nanog and delayed activation of paternal genes in preimplantation embryos (Gu et al., 2011). These findings demonstrate that demethylation of the paternal genome in zygotes is initiated by Tet3-mediated conversion of 5mC to 5hmC and is critical for proper activation of genes essential for embryo development and pluripotency. However, it was argued that a passive demethylation pathway, reliant on DNA replication, was also involved in removal of methylation marks in the paternal pronuclei (Guo F. et al., 2014; Shen et al., 2014). Blocking DNA replication, using a DNA replication inhibitor, maintained DNA methylation level of paternal pronuclei at the same level of sperm DNA, even under Tet3-mediated 5hmC mark formation (Guo F. et al., 2014; Shen et al., 2014). Although the involvement of TET3 on initiating DNA demethylation is widely accepted, a study demonstrated that newly formed 5mC marks via *de novo* methylation are also targeted TET3 and converted into 5hmC (Amouroux et al., 2016). These findings illustrate methylation reprogramming of the paternal genome is a complex process which involves active and passive demethylation and *de novo* methylation processes.

Although deficiency of maternal Tet3 results in sublethality in mice, it is due to haploinsufficiency rather than impaired 5mC oxidation (Inoue et al., 2015). Mouse embryos that bypassed Tet3-mediated 5mC oxidation develop to term normally, and demethylation of paternal genome proceeds as they reached to blastocyst stage, suggesting an existence of compensatory mechanism for defective 5mC oxidation during preimplantation development (Inoue et al., 2015). In particular, Tet1 and Tet3 are on the same branch of the evolutionary tree (Liu D. et al., 2019) and have a similar gene structure (Figure 1). Tet1 expression is relatively lower than Tet3 in developing embryos before the blastocyst stage, but Tet1 protein is apparently detected in early stages such as 2-cell embryos (Kang et al., 2015). In Tet1/3 double knockout embryos strong 5hmC loss and increase of 5mC is observed at 8-cell embryos (Kang et al., 2015). Furthermore, the loss of 5hmC is increased in Tet1/2/3 deficient embryos compared to Tet3 deficient embryos in paternal pronuclei at the zygote stage, suggesting that Tet1 and Tet2 are involved in active demethylation of the paternal genome (Arand et al., 2022). Indeed, Tet1/2 deficiency reduces the levels of 5hmC and 5caC in paternal pronuclei compared to wild-type embryos, implying a distinct role for Tet1 and Tet2 in the sequential oxidation of 5mC to 5caC (Arand et al., 2022). Connection between the loss of 5mC in paternal pronuclei and TET3-driven active demethylation is highly established; however, mechanistic actions underlying the changes remains elusive. In addition to 5hmC, 5fC, and 5caC appear in zygotes concurrently with the loss of 5mC, suggesting that 5hmC is further oxidized potentially by TET3 before cleavage (Inoue et al., 2011). The three 5mC derivatives (5hmC, 5fC, and 5caC) can be direct targets for base-excision repair (BER) pathways mediated by thymine DNA glycosylase (TDG) (He et al., 2011; Maiti and Drohat, 2011). However, depletion of maternal TDG had no effect on zygotic 5fC and 5caC levels (Guo F. et al., 2014), suggesting that TDG is dispensable for paternal pronuclear demethylation. Further studies are necessary to clarify the subsequent demethylation process that reverts oxidized 5mC to unmethylated cytosine because TDG is not consistently detectable in zygotes (Hajkova et al., 2010).

### 4.2 Active and passive demethylation of maternal genome

In contrast to the paternal genome, 5mC in the maternal pronucleus is not targeted by TET3 and rather protected from active demethylation in zygotes (Wossidlo et al., 2011). A maternal factor, STELLA is known to play a key protective role against TET3-mediated 5mC oxidation in maternal pronucleus (Wossidlo et al., 2011; Nakamura et al., 2012). The STELLA, also known as PGC7 and DPPA3, is essential for embryo viability and is a nuclear polypeptide that is highly expressed in PGCs, oocytes, and pluripotent cells (Sato et al., 2002; Bowles et al., 2003). The mating of heterozygous *STELLA*-mutant mice resulted in the birth of STELLA-null offspring without developmental defects (Payer et al., 2003; Bortvin et al., 2004). However, embryos derived from oocytes of STELLA-deficient females were arrested during early cleavage, mostly around the 4-cell stage (Payer et al., 2003; Bortvin et al., 2004). The developmental abnormality was due to the lack of maternally inherited STELLA in the oocytes (Payer et al., 2003; Bortvin et al., 2004). It was suggested that STELLA likely protected the maternal genome from demethylation by localizing to the maternal pronucleus in zygotes, although mechanistic actions of the *STELLA* was unknown (Nakamura et al., 2007).

The discovery of 5hmC and TET proteins in mammals has provided clues to the actions of STELLA. Indeed, STELLA deficiency resulted in TET3-mediated 5hmC accumulation in the maternal pronucleus, demonstrating that STELLA was an important maternal factor for protecting the genome of maternal pronucleus against TET3-mediated demethylation (Wossidlo et al., 2011; Nakamura et al., 2012). It has been demonstrated that the protective role of STELLA is determined by its interaction with demethylated histone H3 Lys9 (H3K9me2), a histone methylation mark enriched only in the maternal pronucleus (Nakamura et al., 2012). STELLA preferentially binds to the maternal genome harboring H3K9me2 marks in zygotes and alters chromatin configuration, thus preventing TET3 binding and inhibiting TET3-mediated 5mC oxidation (Nakamura et al., 2012; Bian and Yu, 2014). A recent study demonstrated that the presence of maternal STELLA sequestered UHRF1, a cofactor of DNMT1, from nuclei of oocytes to protect against hypermethylation (Li Y. et al., 2018). A deficiency in STELLA leads to increased methylation levels in metaphase II oocytes and accumulation of 5hmC, presumably due to a higher availability of 5mC that converts into 5hmC (Li Y. et al., 2018; Han et al., 2019). These findings support the interaction and competition between STELLA and TET3 to steer proper demethylation of the maternal genome upon fertilization.

Despite the protective role of STELLA, demethylation of maternal DNA is not solely mediated by replication-dependent dilution. Maternal genome also partially undergoes Tet3-mediated active demethylation in mouse embryos. Tet3 protein and 5mC oxidation derivatives including 5hmC and 5fC are also detected in maternal pronuclei (Guo F. et al., 2014; Shen et al., 2014; Wang et al., 2014). In Tet3 knockout zygotes, demethylation of maternal DNA is partially blocked (Guo F. et al., 2014; Shen et al., 2014). The 5mC bases, or its oxidized derivatives, are converted to unmodified cytosines at the majority of demethylated CpG dinucleotides in maternal DNA, independent of DNA replication dilution during development from oocytes to four-cell stage (Guo F. et al., 2014; Wang et al., 2014). The existence of both active and passive demethylation in the maternal genome is apparent; however, no clear demethylation pathway has been identified. A recent study has demonstrated that differential distribution of hemi-5mC is found in active genes of the maternal genome, and it indicates demethylation strategies to regulate gene expression in the maternal genome (Cao et al., 2023).

## 5 The DNA demethylation mechanism is conserved in other species

**FIGURE 2:**
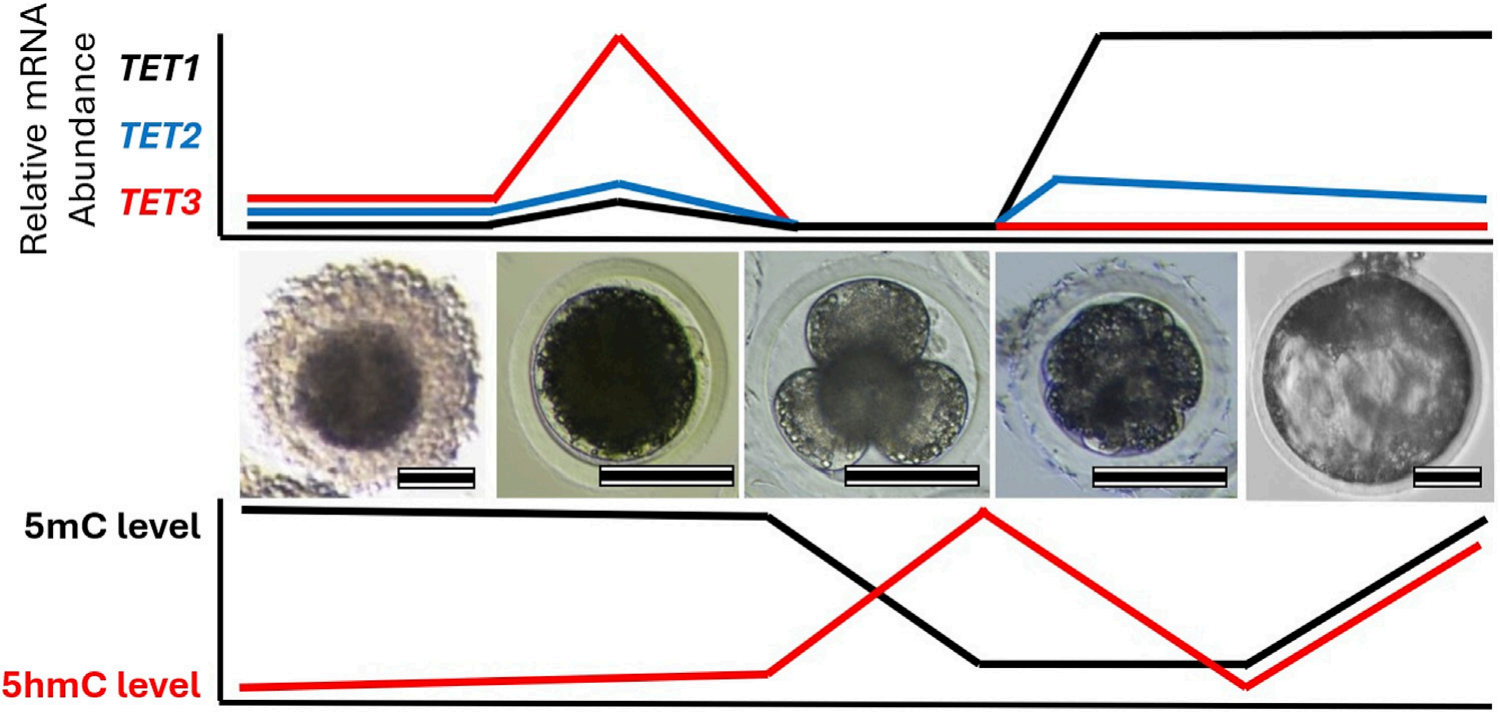
Change in TET family transcript abundance with global 5-methylcytosine (5mC) and 5-hydroxymethylcytosine (5hmC) profiles during early embryo development. Images highlight different stages of porcine oocyte and embryo development: (left-to-right) cumulus-oocyte complex, MII oocyte, 4-cell embryo, compacted morula, and blastocyst. Relative mRNA abundance of each TET family member and DNA methylation levels corresponds to each stage are illustrated. Scale bare represents 100 µm.

Dynamic changes in DNA methylation levels during preimplantation embryo development exhibit similar patterns in mammalian species (Figure 2). For example, a rapid demethylation of the paternal pronucleus occurs in mice, rats, pigs, and cattle (Dean et al., 2001). However, quantitative differences in the level of DNA methylation exist among the species (Kobayashi et al., 2012; Guo H. et al., 2014; Okae et al., 2014; Smith et al., 2014; Ivanova et al., 2020). For instance, differences in DNA methylation levels between species are apparent in cleavage stage embryos. Pig embryos have higher CpG methylation levels (55%) (Ivanova et al., 2020) than mouse embryos (23%–28%) (Altschuler et al., 1985; Smith et al., 2012) at 8–16 cells stage. The higher DNA methylation level in pig embryos decreases dramatically at the blastocyst stage, reaching the lowest level (13%) (Ivanova et al., 2020) compared to other species (19%–29%) (Altschuler et al., 1985; Smith et al., 2012; Smith et al., 2014; Zhu et al., 2018). In contrast to other species, dramatic DNA demethylation is not observed during preimplantation development in sheep (Beaujean et al., 2004; Zhang et al., 2021). During development from gametes to the 16-cells stage, very limited demethylation occurs, then the level of methylation increases until the blastocyst stage (Beaujean et al., 2004; Zhang et al., 2021).

Consistent with the DNA demethylation process, similar expression patterns of TET enzymes are observed in preimplantation embryos of different species (Figure 2). Transcripts of *TET3* are abundant in oocytes and zygotes, while *TET1* is highly expressed in the relatively late-stage embryos in mice, pigs, cows, and sheep (Ito et al., 2010; Gu et al., 2011; Iqbal et al., 2011; Wossidlo et al., 2011; Lee et al., 2014; Jafarpour et al., 2017; Duan et al., 2019; Uh et al., 2020; Uh and Lee, 2022). In mouse zygotes, the role of Tet3 in the demethylation and formation of 5hmC in paternal pronuclei has been identified (Gu et al., 2011; Iqbal et al., 2011; Wossidlo et al., 2011). Similarly, disruption of TET3 impaired 5hmC formation in porcine zygotes and bovine 2-cell embryos (Lee et al., 2014; Zhang et al., 2017; Cheng et al., 2019), suggesting a conserved role of TET3 in the demethylation of fertilized mammalian embryos. Furthermore, the fine-tuning of post-fertilization demethylation by TET3 is required for the regulation of pluripotency gene expression in mammalian embryos. Impaired DNA methylation and expression levels of pluripotency genes due to *TET3* disruption have been detected in developing embryos of mice, pigs, and cows (Gu et al., 2011; Lee et al., 2014; Zhang et al., 2017; Cheng et al., 2019; Uh and Lee, 2022). Genetic ablation of Tet3 leads to perinatal death without pregnancy failure in mice (Gu et al., 2011), but the impact of TET3 deficiency beyond the preimplantation stage has not been reported in other species. TET1, which is enriched in pluripotent stem cells and in the inner cell mass of blastocysts, plays an important role in the regulation of pluripotency. Although Tet1 is dispensable for maintaining pluripotency of mouse embryonic stem cells (Dawlaty et al., 2011), the disruption of *TET1* leads to impaired lineage specification, particularly of the inner cell mass in both mouse and pig blastocysts (Ito et al., 2010; Uh et al., 2020). Conservation of embryonic reprogramming among our domesticated species allows greater understanding of the impact that TET family disruption has on embryo development and their epigenetic landscape (Table 2).

**TABLE 2:** Summary of outcomes of disrupting normal TET family function during early embryo development. The targeted TET family member is denoted on the left along with model species and the type of model generated. The impact of TET family disruption on embryo development, epigenetic landscape, and other relevant outcomes are recorded.

Gene	Species	Model	Outcome	Reference
Tet1	Mouse	Global knockout	Reduced birthweight and increased infertility. Severe neurological defects. Promoter hypermethylation and down-regulation of neural-associated genes	Dawlaty et al. (2011); Yamaguchi et al. (2012); Rudenko et al. (2013); Zhang et al. (2013)
TET1	Swine	siRNA knockdown in zygotes	Reduced blastocyst development. Abnormal methylation patterns and abnormal pluripotency gene expression	Uh et al. (2020)
Tet2	Mouse	Global knockout	Expanded hematopoietic progenitor cell populations inducing myeloid and lymphoid lineage disorders	Li et al. (2011); Moran-Crusio et al. (2011); Ito et al. (2019)
Tet1, 2	Mouse	Global double knockout	Successful generation of double-gene mutant mice by zygote injection. Normal birth-rate and no visible developmental abnormalities. Epigenetic profiles were not examined	Wang et al. (2013)
Tet3	Mouse	Global knockout	Neonatal lethal	Wang et al. (2013); Tsukada et al. (2015)
		Germline-specific knockout	Failure to demethylate paternal pronucleus. Disruption of pluripotency genes. Females have reduced fecundity	Gu et al. (2011)
		Neuronal-specific knockout	Increased anxiety-like behavior and cortisol production; like Cushing’s disease	Antunes et al. (2021)
TET3	Swine	siRNA knockdown in zygotes	Embryo development was not affected, but expression of pluripotency related genes was suppressed	Lee et al. (2014); Uh and Lee (2022)
		Zygotes overexpressed TET3-mutant only containing CXXC domain	Rapid genome-wide demethylation by 2-cell embryo. Upregulation of pluripotency genes in blastocyst	Uh and Lee (2022)
TET3	Cattle	siRNA knockdown in GV oocytes	Disrupted embryo development. Increased 5mC levels and decreased 5hmC levels. Decreased pluripotency-related gene expression	Cheng et al. (2019)
		TET3 overexpressed in SCNT embryo	Improved embryo development. Decreased methylation and subsequent increased transcriptional activity of pluripotency-related genes	Zhang et al. (2020)
TET3	Goat	siRNA knockdown in MII oocytes	Disruption of DNA demethylation at 2-cell and 4-cell stage. Decreased pluripotency gene expression	Han et al. (2018)
		TET3 overexpressed in SCNT embryo	Improved embryo development, pregnancy rates, and birth rates. Prevented hypermethylation of pluripotency gene promoters	Han et al. (2018)

## 6 Long-term health effects due to TET enzyme disruption

Disruption of epigenetic machinery is known to be linked to several neurodevelopmental and growth abnormalities in the clinic. For example, immunodeficiency-centromeric instability-facial anomalies (ICF) syndrome and Tatton-Brown-Rahman syndrome are caused by mutations in *DNMT3B* and *DNMT3A*, respectively (Vukic and Daxinger, 2019; Kumps et al., 2023). Patients with either syndrome show signs of epigenetic abnormalities. There is global hypomethylation of the genome in patients with Tatton-Brown-Rahman syndrome (Kumps et al., 2023) while regions surrounding the centromeres are hypomethylated in patients with ICF syndrome (Vukic and Daxinger, 2019). Beck-Fahrner syndrome is an autosomal dominant disorder with mutations in the catalytic domain to reduce TET3 enzymatic activity (Beck et al., 2020; Fahrner, 2023). Individuals suffering from Beck-Fahrner syndrome commonly have intellectual disability, autistic traits, movement disorders, hypotonia, and facial dysmorphism (Beck et al., 2020; Fahrner, 2023). There are no consensus diagnostic criteria for Beck-Fahrner syndrome except modifications to the *TET3* gene (Fahrner, 2023).

No other genetic diseases are known to have a direct link to mutations in the TET family. However, there are clinical cases that are rooted to abnormal TET activity or somatic mutation of TET family. These clinical cases are difficult to pinpoint clear association of abnormal TET family to their pathogenesis; however, animal studies indicate potential outcomes if the level of TET family is misregulated (Table 2). Mice with *Tet1* KO had a reduction in birthweight and increased infertility (Dawlaty et al., 2011). Reduced follicle size and follicle number contributed to the subfertile phenotype, but progeny was still viable (Dawlaty et al., 2011). It was observed that the ablation of TET1 caused several neurological defects such as reduced spatial learning and impaired short-term memory although their brain weight, neuron number, synaptic activity, or eyesight memory seemed to be normal (Rudenko et al., 2013; Zhang et al., 2013). The disruption of *TET1* caused promoter hypermethylation and subsequent downregulation of genes associated with neural progenitor proliferation, neuroprotection, and mitochondrial function (Rudenko et al., 2013; Zhang et al., 2013). A different phenotype emerges when *TET2* was disrupted. The *Tet2* KO mice had expanded hematopoietic stem cell and progenitor cell populations (Li et al., 2011; Moran-Crusio et al., 2011; Ito et al., 2019), which induced disorders in myeloid and lymphoid cell lineages mimicking chronic myelomonocytic leukemia (CMML) (Moran-Crusio et al., 2011; Ito et al., 2019). Similar phenotypes are seen in the human clinic as several myeloid malignancies have loss-of-function mutations in TET2 (Li et al., 2011). There are fewer functional studies of *TET3* as the knockout mice model appears to be neonatal lethal (Wang et al., 2013; Tsukada et al., 2015). It was demonstrated that a conditional *Tet3* knockout in brain neurons increased anxiety-like behavior and cortisol production that’s similar to Cushing’s disease (Antunes et al., 2021).

Various somatic-cell diseases are associated with disruption of the TET family. For example, it has been shown that TET1 is downregulated in many tumor cell lines (Li et al., 2016). In breast cancer, uterine cancer, or glioblastoma, there is an enrichment of a truncated TET1 isoform that lacks the CXXC domain (Good et al., 2017), thus presumably inhibit normal action of TET1. The promoter methylation of tumor suppressing genes in these cells remained to be high and silenced these genes (Li et al., 2016; Good et al., 2017). Proper regulation of the TET family is necessary to avoid disease onset. It is well established that deregulation of WNT signaling pathway induces early-stage cancer formation (Klaus and Birchmeier, 2008). Normal TET1 expression supports WNT pathway inhibitors that inhibit cell migration, cell division, and epithelial-mesenchymal transition (Duan et al., 2017; Fan et al., 2018; Wu et al., 2019). This disruption of TET2 is common in hematopoietic cancers as its enzymatic activity mediates the formation of immune cells, especially T cells, B cells, and macrophages. Specifically, the loss of TET2 causes hypermethylation in enhancer regions within immune progenitor cells which induces their tumorigenesis (Jiang, 2020). Somatic-cell cancers such as ovarian, head/neck squamous cell carcinoma, gastric, and colorectal cancers have been implicated with TET3 disruption but the mechanistic influence of TET3 remains unclear (Misawa et al., 2018; Cao et al., 2019; Mo et al., 2020).

Abnormal TET family disruption is linked to several neurological diseases found in the clinic. The level of 5hmC is enriched in mammalian brain compared to other tissues (Tahiliani et al., 2009; Globisch et al., 2010; Song et al., 2011). As one of the main regulators of 5hmC, it is not a surprise TET3 is highly present in many brain regions, including the cerebral cortex, hippocampus and cerebellum, and its expression level is stable across different brain regions (Szwagierczak et al., 2010). As aforementioned, mutation in *TET3* causes Beck-Fahrner syndrome where patients present intellectual disability and developmental delay ranging from mild to severe affecting both motor and speech abilities (Beck et al., 2020; Fahrner, 2023). To date, studies using animal models carrying neurons with a disrupted *TET3* gene have reported increased anxiety-like behavior, impaired spatial orientation and short-term memory (Antunes et al., 2021; Antunes et al., 2022; Fan et al., 2022), suggesting potential risks of *TET3*-related neurological disorders in humans. Rare variations of TET2 were often found in patients with early onset Alzheimer’s diseases (AD) and frontotemporal dementia (FTD) (Cochran et al., 2020). Similarly, a mouse model carrying repressed level of *Tet2* accelerated at presenting age-related neurogenic decline and the overexpression of *Tet2* could rescue the abnormalities by protecting the animals from age-related neurodegenerative decline and enhanced their cognitive function (Gontier et al., 2018). Patients with psychosis, such as schizophrenia or bipolar disorders, do have increased expression of TET1 and altered levels of 5hmC in their brain (Dong et al., 2012; Kwon et al., 2018). The level of 5hmC is enriched in various neuronal cells (Kriaucionis and Heintz, 2009; Globisch et al., 2010; Ruzov et al., 2011; Mellén et al., 2012) and maintains for months without further modifications in the brain (Bachman et al., 2014). Unlike 5mC levels, which are relatively consistent across different tissue types (Li and Liu, 2011), the level of 5hmC is highly tissue specific, ranging from 0.03% of all cytosines in the spleen to 0.7% in the brain (Globisch et al., 2010; Li and Liu, 2011). The enrichment of 5hmC indicates the base is more than an intermediate during DNA demethylation process, but rather a stable epigenetic mark that regulates activity of nervous system (Gontier et al., 2018).

**FIGURE 3:**
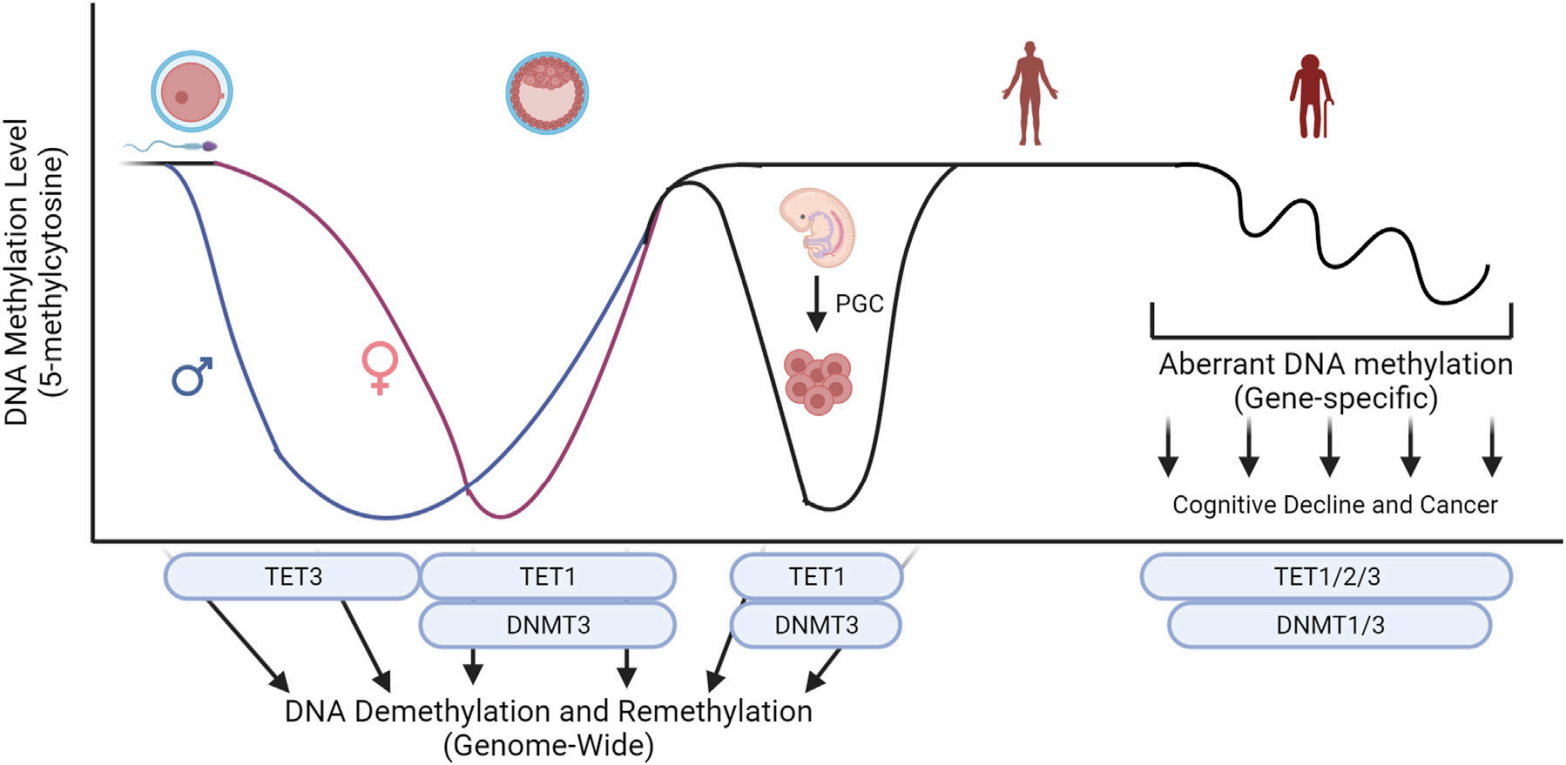
Key epigenetic machinery during embryo development and their influence on global levels of 5-methylcytosine. Conversion of 5-methylcytosine by the TET family and *de novo* methylation by DNMT3 sculpture the level of DNA methylation during development. Abnormal global epigenetic reprogramming during development or somatic mutations on key epigenetic regulators can cause diseases including neurological disorders and cancers. Created with BioRender.com.

Improper maintenance of DNA methylation is linked to clinical diseases, and abnormal levels of *TET* family are often associated with the diseases. Abnormal global epigenetic reprogramming during development or somatic mutations on key epigenetic regulators can cause diseases including neurological disorders and cancers (Figure 3). Understanding mechanistic actions of *TET* family using cell and animal models will lead to the development of clinical interventions that can be used to design customized treatment options against these epigenetic disorders.

## 7 Influence of assisted reproductive technology on embryonic reprogramming

Assisted reproductive technologies (ART) are widely used in the clinic and as a technology to sustain food production around the world. In the clinic, it is estimated that over eight million children have been born via ART in western countries to date, and that approximately 6% of births in Europe in 2014 were conceived through ART (Wennerholm and Bergh, 2020). Although commonly applied, the long-term impact of ART has not been clear. It is estimated that children born using ART have a 1.4 to 1.9-fold higher risk of birth defects than children born naturally (Olson et al., 2005; El-Chaar et al., 2009; Giorgione et al., 2018). A retrospective study also states ART-pregnancies has a risk factor for epigenetic diseases three times higher than normal pregnancy (Uk et al., 2018). Beckwith-Wiedemann and Silver-Russel syndrome, both of which occur due to disruption of epigenetic imprints, have a 10-fold greater frequency in ART-conceived children compared to natural conception (Hiura et al., 2012). In addition, the methylation of CpG sites in placental and umbilical appears to be dysregulated (Katari et al., 2009).

Due to the lack of *in vitro* models for human implantation and placentation, studies in animal models reflect how ART may impact embryo quality and subsequent development. When compared to embryo development *in vivo*, ART-derived blastocysts contain a lower total number of cells and a reduced ratio of inner cell mass to trophectoderm cell numbers (Macháty et al., 1998; Yoshioka et al., 2002; Canovas et al., 2017). Similar to humans, the DNA methylomes are disrupted in domesticated species by the *in vitro* culture system (Barrera et al., 2017; Mani and Mainigi, 2018; Salilew-Wondim et al., 2018). In addition, ART-derived embryos would have a relatively hypermethylated global genome (Deshmukh et al., 2011; Cao et al., 2014; Zhang et al., 2016; Han et al., 2018). ART-induced epigenetic abnormalities are also well-documented in rodent models (Doherty et al., 2000; Market-Velker et al., 2010; Market Velker et al., 2012; Vrooman et al., 2022). Aberrant methylation, in ART-derived embryos, often occurs at promoter and intergenic regions (Canovas et al., 2017; Cao et al., 2020), but some imprinted regions can be disrupted as well (Canovas et al., 2017). Interestingly, a large proportion of embryos generated from somatic cell nuclear transfer (SCNT) present epigenetic abnormalities because of incomplete epigenetic reprogramming of donor cell DNA (Rideout et al., 2001; Mann and Bartolomei, 2002; Bonk et al., 2007). Embryos derived from SCNT have greater global DNA methylation than other ART-derived embryos (Kwon et al., 2008; Huan et al., 2014). In addition, SCNT-derived embryos that are arrested during early cleavage stages have greater genomic methylation than those that developed into blastocyst (Gao et al., 2018). The hypermethylation of SCNT-embryonic genome appears to disrupt transcription of genes essential for zygotic-genome activation, such as Dppa2 and Dppa4 (Cao et al., 2020). Aberrant *de novo* methylation has been linked to the transcriptional dysregulation of SCNT-embryos (Song et al., 2017; Gao et al., 2018). However, embryonic reprogramming of SCNT-embryos appears to be locus-specific (Zhang et al., 2016), implicating multiple epigenetic modulators being dysregulated.

It is not clear if abnormal activity of epigenetic modulators, such as TET family, in ART-embryos is directly responsible for the epigenetic abnormalities. However, since expression of these pluripotency factors are decreased in cattle and pig *in vitro* embryos (Cheng et al., 2019; Uh et al., 2020), and *TET* family is directly related to pluripotency (Costa et al., 2013), presumably, activity of *TET* family is associated with the epigenetic abnormalities in ART-embryos. Stress from *in vitro* culture systems often disrupt reprogramming ability of the TET family and other epigenetic modulators. Mouse and bovine embryos derived from *in vitro* fertilization have abnormal level of the TET family as it develops into a blastocyst (Chu et al., 2021). Additionally, epigenetic changes occur when embryos are removed from a low oxygen environment (∼5%) (Gaspar et al., 2015; Skiles et al., 2018). Bovine embryos exposed to atmospheric levels oxygen (∼20%) carry disrupted transcript level of epigenetic modulators such as polycomb repressor complex, histone methyltransferases, histone demethylases, and TET family enzymes (Skiles et al., 2018). Cryopreservation of mouse and bovine oocytes results in a global abnormality in 5mC and 5hmC (Chen et al., 2016; Fu et al., 2019). These abnormalities may be corrected by functioning TET family enzymes. The presence of functional *TET3* greatly influences the transcript abundance of pluripotent factors, such as *NANOG* or *OCT4*, in domesticated species (Gu et al., 2011; Skiles et al., 2018; Cheng et al., 2019; Uh et al., 2020). For instance, cattle and goat embryos that overexpress *TET3* have been shown to perform much better during *in vitro* culture (Han et al., 2018; Zhang et al., 2020). The inclusion of embryokines (such as FGF2 and LIF) does not rescue TET enzyme suppression by *in vitro* culture (Chu et al., 2021), but the activity of available TET enzymes has been stimulated by Vitamin C supplementation which improved 5mC and 5hmC levels (Zhang et al., 2016; Fu et al., 2019; Zhang et al., 2019; Chu et al., 2021). It may be fruitful to investigate how *in vitro* culture systems influence the function of the TET enzymes or their cofactors as the TET family appear to be major contributors to ART-derived embryo success.

Use of embryos produced via ART is an important part of sustaining livestock productivity. Elite genetics can be rapidly introduced into the production system and ART-embryos between elite animals can enhance genetic improvement (Vishwanath, 2003; Sirard, 2018). Although successful animal production from ART-embryos, even at industry scale, is possible, the ART-embryos and subsequent animals may have epigenetic abnormalities. For example, *in vitro* conceived cattle often suffer from large offspring syndrome (LOS), which is caused by aberrant expression of insulin-like growth factor 2 receptor (*IGF2R*) due to abnormal imprinting errors (Urrego et al., 2014). In addition, loss-of-imprinting was detected in ART-induced LOS fetus tissues (Chen et al., 2015). Oocytes derived via *in vitro* maturation is known to have differentially methylated regions, compared to their *in vivo* counterparts (Jiang et al., 2018). Several other developmentally important genes have been shown to be disrupted in *in vitro* bovine embryos, such as X (inactive)-specific transcript (*XIST*) and insulin-like growth factor 2 (*IGF2*) (Urrego et al., 2014). As mentioned earlier, the TET-mediated epigenetic reprogramming is conserved in many species, including livestock. Utilizing mechanistic actions of epigenetic modulators such as the TET family will assist us to correct epigenetic abnormalities associated with ART-embryos.

## 8 Conclusion

Proper epigenetic reprogramming upon fertilization is coordinated by the *TET* family and is essential for successful development. These enzymes are indispensable as several neurological disorders and cancers are associated with the malfunction of TET enzymes. While species differences do exist, the role of *TET* family is highly conserved among different mammals. Understanding mechanistic actions of the TET family can provide clues to improve the well-being of individuals suffering from epigenetic disorders and maintain epigenetic integrity of embryos produced via assisted reproductive technologies.
